# To chat or not to chat: how osteopathic students utilize artificial intelligence platforms in pharmacology education

**DOI:** 10.3389/fphar.2026.1930362

**Published:** 2026-10-27

**Authors:** Lyla Barr, Sammy Bakeer, Brooke Bemowski, Sarena Orio, Naunihal Zaveri

**Affiliations:** 1 First Year Osteopathic Medical Student, Baptist University College of Osteopathic Medicine, Memphis, TN, United States; 2 Pharmacology Faculty, Baptist University College of Osteopathic Medicine, Memphis, TN, United States

**Keywords:** AI platforms, artificial intelligence, osteopathic medical education, pharmacology education, student learning, queries

## Abstract

**Introduction:**

The rapid development of artificial intelligence (AI) tools has led to their expanding use in academia, medicine, and medical education. However, the quality, accuracy, source reliability, and integration of osteopathic principles in AI‐generated content for pharmacology education remain insufficiently explored. Our goals were to use AI tools to generate pharmacology content, which we could critically evaluate for quality and accuracy, while exploring the capabilities and limitations of the outputs. The content we asked AI to generate was based on pharmacologically important concepts for osteopathic physicians in training, with the application of osteopathic philosophy included in some of the queries.

**Methods:**

Three leading AI platforms, namely, Consensus Deep, Perplexity Pro, and ChatGPT Plus, were evaluated using three pharmacologically relevant queries. Each query was input into each platform, and the outputs were evaluated for quality, credibility, and source validity. The AI platforms we used required a paid subscription.

**Results:**

Our results showed that Consensus Deep consistently provided output that included peer-reviewed journals and integrated osteopathic principles into its responses. Perplexity Pro provided information with references; several references included websites or social media posts, and when the prompt was refined and repeated, the output was limited and required additional purchase for further usage. ChatGPT Plus provided output which was patient-centered; when prompted for references, it provided references which had to be assessed for quality or credibility.

**Discussion:**

Our study results indicate that generative AI platforms are a very useful tool for pharmacology and osteopathic medical education, although the use of the AI-generated content requires learners to critically evaluate the outputs for integrity, quality, and accuracy. In addition, there are currently limited data across AI platforms that include osteopathic principles and philosophy in pharmacology and therapeutics, and future work should be directed toward increasing scholarly research and publications in the fields of osteopathic medicine, pharmacology learning, and AI use.

## Introduction

Artificial intelligence (AI) is a rapidly developing technology widely used for data management and customer support and now is increasingly used in medicine and education. Turing (1950) proposed the question “Can machines think?”, and by 2018, the launch of the original generative pre-trained transformer (GPT) by OpenAI started the development of large language models (LLMs) as advanced forms of machine learning. This was followed by the creation of ChatGPT in 2022 (OpenAI, 2022), and currently, there are several AI platforms that serve as generative AI tools that can provide text, images, video, or audio content upon being queried using “prompts.” Using natural language processing (NLP) algorithms, these platforms can provide content that is unique, of good quality, easy-to-read, and often broad in scope due to its ability to scan large volumes of material. These tools have been used in medicine and medical education to generate practice questions, simulate standardized patients, and provide feedback in teaching strategies (Yu et al., 2025; Dave et al., 2023). Despite its usefulness, the reliability of the responses from AI platforms can fluctuate, often resulting in “hallucinations,” providing false answers or generating fictional sources of information which can adversely affect patient care (Athaluri et al., 2023) and student learning.

Pharmacology is a foundational biomedical science that is necessary for lifelong practice for physicians, to perform therapeutic management that is safe, effective, and patient-centered. In a future where AI will be ubiquitous, students’ AI literacy and ability to critically evaluate AI-generated content and understand its capabilities and limitations for pharmacology learning in medical education will be of tremendous importance (Guilding et al., 2026).

Our goal in this study was to explore how AI can be used to learn pharmacology concepts for osteopathic medical education. Tenets of the osteopathic philosophy include the following: the person is a unit of body, mind, and spirit; the body is capable of self-healing; structure and function are reciprocally interrelated; and finally, rational treatment is based on the first three principles (American Osteopathic Association, 2018). Osteopathic manipulative treatment (OMT) was derived from these tenets, and these elements are underrepresented in LLMs and in clinical algorithms due to the limited osteopathic literature in training datasets (Newhardt et al., 2026). It is therefore imperative that osteopathic learners critically appraise AI outputs and recognize biases, such as the prioritization of pharmacologic interventions, including opioids or prescription drugs, over the benefits of combining OMT with cognitive behavioral therapy for chronic pain (Hussein et al., 2019). A recent study from an osteopathic medical school demonstrated that students use AI tools to a significantly higher extent than faculty for studying, generating, and reviewing exam questions and completing written assignments (Panta et al., 2026), although they were less likely to receive formal training in the use of these tools than faculty. Another study exploring the use of AI in pharmacology teaching included asking students to critique responses generated using Bing, ChatGPT 3.5, and Google Bard (Swanson, 2024), although not all pharmacy or medical school curricula include AI literacy.

AI is currently being integrated into numerous medical specialties, including radiology, pathology, and ophthalmology, and a meta-analysis showed that research on AI in nursing practice and nursing education is in the early stages of development (Amiri et al., 2024; Ito and Ikehara, 2024). Similarly, in osteopathic medical education, the use of AI tools by medical students and the limitations of these tools are in early stages of integration and remain underexplored. A recent study evaluated the accuracy of ChatGPT, Copilot, and Google Gemini in answering questions related to cardiovascular (CV) pharmacology, showing that ChatGPT-4 demonstrated the highest accuracy in addressing CV pharmacology questions, followed by Copilot and then Gemini (Salman et al., 2025).

In this study, we aim to examine the reliability, quality, and accuracy of AI outputs and integration of osteopathic principles (when possible) of different AI platforms which are queried for pharmacology content. Our approach involved choosing three pharmacology-relevant topics to use as prompts, which would be input as queries into different AI platforms, and analyzing the outputs. After several rounds of discussion and literature searches, we finalized three queries related to important pharmacology concepts to use in this study:

1. What are the risks of the long-term use of GLP-1 receptor agonists for weight loss or diabetes?
2. Does the application of OMT reduce or supplement the use of opioids for chronic pain management?
3. What are prevention measures for adverse drug reactions in geriatric populations with polypharmacy using a whole-person approach?

Three AI platforms were selected based on their frequency of use by the general population and medical students, as well as the breadth of the LLMs that they cover as independent research and search engines. The above three queries were used as prompts which were input into the three platforms, and we collected, examined, and analyzed the outputs. Our specific aims were to critically evaluate the outputs, verify whether the information was generated from credible sources, and examine whether osteopathic principles were included in the findings especially when it was not explicitly included in the query.

## Methods

Three AI platforms were selected for prompt input and analysis: Consensus Deep, Perplexity Pro, and ChatGPT Plus. Data collection occurred between April 2026 and June 2026, during which period, the monthly subscription costs for these platforms were $65 USD for Consensus Deep, $17 USD for Perplexity Pro, and $20 USD for ChatGPT Plus. Each prompt (Query) had three researchers assigned to it and was queried at least two times on each platform at different times on different days to follow guidelines stated in Frontiers: AI in Research, 2026. Query 1 was assigned to SB, LB, and BB; Query 2 was assigned to SB, LB, and SO; Query 3 was assigned to BB, SO, and NZ. Prompt wording was refined after a few trials, and the same final prompt was used by all researchers for consistency. When follow-up questions were asked for any query, all researchers maintained consistency by asking the same questions for that query. The faculty member (NZ) independently conducted the search for each query using each platform.

Outputs from each query were examined and evaluated using the following criteria: whether references were provided without prompting, number of references provided, publication year of each reference, and reference/study type (systematic review, meta-analysis, randomized controlled trial, *etc.*); whether the reference was related to the initial query; if the source was peer-reviewed; and if there was an osteopathic affiliation of authors or institution. After initial evaluation, duplicate references and non-peer reviewed publications were excluded from the final number of references reported for each platform.

To evaluate the credibility and verifiability of literature references returned by each AI platform, four student researchers (LB, SB, BB, and SO) independently scored the nine platform-by-query outputs; LB scored all nine combinations (of query + platform), while the other three students scored six combinations each (for the query they were assigned to). For credibility, a score of 1 (low credibility) was given for information that came from a blog, commercial website, news article, poster/presentation, unreviewed PDF, or opinion piece; a score of 2 (moderate) was assigned when the evidence was from a reputable medical/educational source, narrative review, academic medical center page, NCBI bookshelf/StatPearls chapter, or professional organization informational page that is not classified as original research or primary study findings; a score of 3 (high) was assigned where references were peer-reviewed original research articles, systematic reviews, meta-analyses, randomized-controlled trials, clinical practice guidelines, or government/major professional organization evidence reports. For verifiability, a score of 1 (low) was assigned when the listed reference did not exist, was fabricated, unclear, or did not support the claim made in the response; a score of 2 (moderate) was assigned when the reference existed, but details were limited, and/or claims were hard to trace; a score of 3 (high) was assigned when the reference exists, and the authors, date, and claims are clear and traceable. Reference credibility and verifiability scores from each platform were averaged and recorded. Discrepancy in scores required reevaluation by all members and further analysis of a reference and its components. Because each researcher submitted the query independently, the set and number of references returned varied across researchers even for the same platform and query.

Because a different subset of three researchers (drawn from a pool of four) rated each output, inter-rater reliability was assessed using a one-way random effects intraclass correlation coefficient (ICC), Case 1, following Shrout and Fleiss (1979) and consistent with the guidelines of Koo and Li (2016). This model does not require the same raters to score every output, making it appropriate for the rotating-rater design used here. ICC(1,1) estimates the reliability of a single rater’s score, and ICC(1,k) estimates the reliability of the averaged score across the three raters assigned to each output (k = 3); the latter corresponds to the reference credibility and verifiability values reported for each platform-by-query output. ICCs were computed separately for credibility and verifiability across the nine outputs, with 95% confidence intervals derived from the F-distribution of the underlying one-way analysis of variance. ICC values were interpreted using the benchmarks of Koo and Li (2016): < 0.50, poor; 0.50–0.75, moderate; 0.75–0.90, good; >0.90, excellent.

Responses were evaluated for osteopathic affiliation based on the following criteria: whether the first or second author was a Doctor of Osteopathic Medicine (D.O.), whether the researchers or institution was affiliated with an osteopathic school, or whether the research was published in an osteopathic journal. These criteria are consistent with established descriptions of “COM-affiliated research,” which encompasses research conducted by DO-trained researchers or through accredited osteopathic medical schools (American Association of Colleges of Osteopathic Medicine, 2026).

## Results

To evaluate the educational utility of the selected AI platforms, three final optimized prompts were used to query the platforms, and outputs were analyzed. The responses generated using Consensus Deep, Perplexity Pro, and ChatGPT Plus were systematically assessed and scored to determine reliability, clinical accuracy, and source validity as it relates to pharmacology and osteopathic education.

Query 1: “What are the risks of long-term use of GLP-1 receptor agonists for weight loss or diabetes?”

Table 1 provides results from Query 1 and lists the following: osteopathic approach, number of references, average score for credibility, percentage of peer-reviewed references, average score for reference reliability, osteopathic affiliation, and average number of repeat references.

**TABLE 1:** Query 1 “What are the risks of long-term use of GLP-1 receptor agonists for weight loss or diabetes?” prompted into ChatGPT Plus, Perplexity Pro, and Consensus Deep. Numbers in parentheses are scores for each individual researcher. Reference credibility and verifiability were scored as described in methods, and scores of three researchers were averaged.

Query #1	ChatGPT Plus	Perplexity Pro	Consensus Deep
Osteopathic approach in response?	No	No	No
Number of references	Range: 11–18 Mean: 15	Range: 13–29 Mean: 23	Range: 20–20 Mean: 20
Average reference credibility	2.38 (1.80, 2.55, 2.78)	1.95 (2, 1.75, 2.11)	2.8 (2.65, 3, 2.75)
Percent of peer reviewed references	49% (9/15, 6/11, 6/18)	39% (5/13, 9/28, 9/19)	100% (20/20, 20/20, 20/20)
Average reference verifiability	2.83 (2.80, 2.81, 2.89)	2.4 (2.05, 2.46, 2.69)	3 (3, 3, 3)
Osteopathic affiliation?	No (1/15, 0/11, 2/18)	No (1/13, 4/28, 1/19)	No (0/20, 0/20, 0/20)
Average number of repeat sources	1 (0, 0, 4)	3 (0, 1, 8)	0 (0,0,0)

ChatGPT Plus provided a range of 11–22 references with an average of 15, with 1 repeat. The platform did not provide references automatically and required secondary prompting to list references. Average credibility and verifiability for the sources were 2.38 and 2.83, respectively. The percentage of peer-reviewed references was 49; there was minimal osteopathic approach or affiliation in the listed references or the response. The clinical content of the generated text identified a comparable number of affected organ systems relative to other models. Notably, most of the pharmacological references specifically addressed semaglutide rather than providing a broader analysis of the GLP-1 receptor agonist class or other drugs in the class (for example, liraglutide or dulaglutide).

Perplexity Pro provided a range of 13–29 references with an average of 23, with 3 repeats; these were provided automatically without a need for prompting. This platform yielded a unique profile, including a wide variety of media, such as scientific articles, websites, blogs, and social media content (YouTube videos and Facebook posts). Average credibility and verifiability scores were 1.95 and 2.4, respectively. The percentage of peer-reviewed references was 39; there was a minimal osteopathic approach or affiliation in the listed references or the response. When the platform was prompted further, continued inquiry within the same thread triggered prompts for the user to purchase additional credits to continue. This limitation could be bypassed by starting a new query; however, the context for ongoing analysis was lost.

Consensus Deep provided 20 references for each researcher with no repeats. Average credibility and verifiability scores were 2.8 and 3, respectively. The percentage of peer-reviewed references was 100; there was minimal osteopathic approach or affiliation in the listed references or the response. The platform demonstrated a transparent data-processing approach, explicitly stating the number of articles that were being retrieved, found eligible, and ultimately included in the output. Each reference cited was accompanied by a detailed description, including article title, access link, article type, publication information, quality ratings, and a brief description of the contribution of the reference article to the generated output.

Query 2: “Does the application of OMT reduce or supplement the use of opioids for chronic pain management?”

Table 2 provides results from Query 2, listing the same parameters being tested as in Table 1.

**TABLE 2:** Query 2 “Does the application of osteopathic manipulative treatment reduce or supplement the use of opioids for chronic pain management?” prompted into ChatGPT Plus, Perplexity Pro, and Consensus Deep. Numbers in parentheses are scores for each individual researcher. Reference credibility and verifiability were scored as described in methods, and scores of three researchers were averaged.

Query #2	ChatGPT Plus	Perplexity Pro	Consensus Deep
Osteopathic approach in response?	Yes	Yes	Yes
Number of references	Range: 4–28 Mean: 14	Range: 14–23 Mean: 18	Range: 20–26 Mean: 22
Average reference credibility	2.59 (2.75, 2.32, 2.7)	1.72 (1.91, 1.67, 1.58)	2.70 (2.69, 2.75, 2.65)
Percent of peer reviewed references	70% (16/25, 7/10, 3/4)	42% (11/22, 3/6, 3/12)	92% (18/20, 19/20, 24/26)
Average reference verifiability	2.91 (2.84, 2.9, 3)	2.52 (2.64, 2.5, 2.42)	2.95 (2.95, 2.95, 2.96)
Osteopathic affiliation?	Yes (21/25, 7/10), No (1/4)	Yes (17/22, 6/6, 9/12)	Yes (19/20, 18/20, 14/26)
Average number of repeat sources	0 (0,0,0)	4 (0, 10, 2)	0 (0,0,0)

ChatGPT Plus provided a wide range of references in response to this query, ranging from 4–28 with an average of 14, with no duplicates. Average credibility and verifiability scores were 2.59 and 2.91, respectively. The percentage of peer-reviewed references was 70, and there was an osteopathic affiliation in the references. The response framed OMT as an adjunctive, nonpharmacological therapy rather than a guaranteed replacement for opioids in chronic pain management. However, the overall response remained relatively brief and broad in scope by suggesting that OMT could supplement opioids and may help reduce opioid reliance when managing chronic pain (Dowell et al., 2022). No evidence‐supported claims were provided from this platform.

Perplexity Pro provided a range of 14–23 references with an average of 18; four of these were duplicates. Average credibility and verifiability scores were 1.72 and 2.52, respectively. The percentage of peer-reviewed references was 42, and there was an osteopathic affiliation in the references and approach of the responses. The generated output was a concise summary that emphasized OMT as a potentially opioid-sparing adjunct rather than a definitive substitute for opioid therapy in chronic pain management. This information was generated from a small number of references that focused primarily on low back pain and clearly differentiated between observed trends and the current lack of large, high-quality trials directly quantifying opioid dose reduction. Overall, it provided a narrative synthesis that was clinically useful for framing the role of OMT in chronic pain management.

Consensus Deep provided a range of 20–26 references with an average of 22, with no repeats. Average credibility and verifiability scores were 2.70 and 2.95, respectively. The percentage of peer-reviewed references was 92, and there was an osteopathic affiliation in the references and approach of the responses. The output generated was well referenced, consistently prioritizing peer-reviewed journal articles and clinical research. Citations were automatically provided within the narrative, allowing for efficient verification without additional prompting. The response stated that OMT was a plausible opioid-sparing adjunct, highlighting several controlled and observational trials in which OMT was associated with decreased postoperative opioid consumption, as well as with reduced opioid use among patients with chronic lower back pain. Overall, the generated content was organized, academically grounded, and written in a way that connects research findings to clinical significance.

Query 3: “What are prevention measures for adverse drug reactions in geriatric populations with polypharmacy using a whole person approach?”

Table 3 provides results from Query 3, listing the same parameters being tested as in Tables 1, 2.

**TABLE 3:** Query 3 “What are prevention measures for adverse drug reactions in geriatric populations with polypharmacy using a whole person approach?” prompted into ChatGPT Plus, Perplexity Pro, and Consensus Deep. Numbers in parentheses are scores for each individual researcher. Reference credibility and verifiability were scored as described in methods, and scores of three researchers were averaged.

Query #3	ChatGPT Plus	Perplexity Pro	Consensus Deep
Osteopathic approach in response?	No	No	No
Number of references	12, 6, 17 Average: 12	11, 10, 9 Average: 10	20, 18, 19 Average: 19
Average reference credibility	2.38 (2.4, 2.5, 2.24)	1.96 (2, 2.1, 1.8)	2.37 (2.4, 2.28, 2.42)
Percent of peer reviewed references	14% (0/10, 0/4, 7/17)	30% (3/11, 3/10, 3/9)	98% (20/20, 17/18, 19/19)
Average reference verifiability	2.94 (2.9, 3, 2.88)	2.71 (2.73, 2.6, 2.8)	3 (3, 3, 3)
Osteopathic affiliation?	No (0/10, 0/4, 0/17)	No (2/11, 0/10, 0/9)	No (0/20, 0/18, 0/19)
Average number of repeat sources	1 (2, 2, 0)	1 (0, 1, 0)	0 (0, 0, 0)

ChatGPT Plus provided a range of 6–17 references, with an average of 12; there was one repeat source and only a few were found to be primary scientific studies. Average credibility and verifiability scores were 2.38 and 2.94, respectively. The percentage of peer-reviewed references was 41, and there was a minimal osteopathic approach or affiliation in the listed references or the response. The output for all three researchers listed key prevention measures including the following: medication reconciliation, deprescribing, geriatric prescribing tools such as Beers Criteria, STOPP/START criteria, dose adjustment, nonpharmacologic interventions, caregiver involvement, functional assessment, and care coordination.

Perplexity Pro provided an average of 10 references, with 1 duplicate. Average credibility and verifiability scores were 1.96 and 2.71, respectively. The percentage of peer-reviewed references was 30, and there was a minimal osteopathic approach or affiliation in the listed references or the response. The key prevention measures listed by Perplexity were similar to those provided by ChatGPT above, and while it provided greater detail, further prompting required an additional purchase of credits to continue the thread.

Consensus Deep provided 18–20 references with an average of 19, with no duplicates. Average credibility and verifiability scores were 2.37 and 3.0, respectively. The percentage of peer-reviewed references was 98, and there was a minimal osteopathic approach or affiliation in the listed references or the response. In addition to the standard Beers and STOPP/START criteria, this platform identified additional prescribing tools and clinical criteria not mentioned by the other models. Similar to Perplexity, the output contained more information regarding the whole-person approach than did ChatGPT. Interpretation of the osteopathic approach in this platform was highly technical, focused on individualized patient goals and prescribing modifications.

To account for variation among raters for credibility and verifiability scores, intraclass correlation coefficients (ICCs) are reported in Table 4. Inter-rater reliability was good for reference credibility scores, ICC(1,k) = 0.87, 95% CI (0.62–0.97), and excellent for reference verifiability scores, ICC(1,k) = 0.93, 95% CI (0.80–0.98) (Table 4). Single-rater reliability was moderate for credibility, ICC(1,1) = 0.70, 95% CI (0.35–0.91), and good for verifiability, ICC(1,1) = 0.83, 95% CI (0.57–0.95). Both omnibus F-tests were statistically significant [credibility: F (8,18) = 7.94, p < .001; verifiability: F (8,18) = 15.19, p < .001], indicating that between-output variance substantially exceeded within-output (rater) variance for both dimensions.

**TABLE 4:** Intraclass correlation coefficients for reference credibility and verifiability across nine platform-by-query outputs.

Reference dimension	ICC(1,1) (single rater)	95% CI	ICC(1,k) (3-rater avg.)	95% CI	F (8,18)
Credibility	0.698	[0.35, 0.91]	0.874	[0.62, 0.97]	7.94*
Verifiability	0.826	[0.57, 0.95]	0.934	[0.80, 0.98]	15.19*

ICC, intraclass correlation coefficient, one-way random-effects model, Case 1 (Shrout and Fleiss, 1979). ICC(1,1), reliability of a single rater’s score; ICC(1,k), reliability of the mean of k = 3 raters’ scores. CI, confidence interval. *p < .001.

## Discussion

Several AI platforms are used in medical education and research; each has a different purpose it was trained and/or programmed for. The three platforms selected for this study are Perplexity, ChatGPT, and Consensus, chosen for their ability to perform independent research and searches. Perplexity combines LLMs with a live web search to provide detailed answers to queries (Roy, 2025); it can access OpenAI’s GPT models but functions as an independent search engine. ChatGPT is an NLP model that functions to generate human-like responses to user inputs and can be tailored to a specific user via “training” through conversational exchanges (Sable et al., 2024). Consensus is an AI platform to help assist researchers in navigating the scholarly literature to grasp the scientific consensus of research questions input by users (Faix, 2025). Although each platform has a free version, we paid a 1-month subscription for the latest version of Perplexity Pro, ChatGPT Plus, and Consensus Deep for this study.

This comparative evaluation of Perplexity Pro, ChatGPT Plus, and Consensus Deep highlights a high degree of variation in reliability of AI platforms when used for pharmacology and osteopathic medical education. A primary finding of this study was the variation in the source/reference quality across the three queries. Although the exact same query was used as a prompt by three different researchers, the outputs showed variation, leading us to create a scoring system for validating and verifying the references in the outputs. While each query was input by three researchers (which included a faculty coauthor), the faculty member also independently input all queries and compared outputs to those obtained by the students, although they did not score the references for validity. For each query, Consensus Deep displayed the most consistent and academically focused output, demonstrated the highest quality and most rigorous criteria of its references, and consistently provided research papers that were almost always peer-reviewed, had high verifiability scores, and had high reference credibility scores. This platform also provided additional data regarding each citation. In contrast, Perplexity Pro utilized a broad spectrum of media sources, some of which were opinion-based, while most were not peer-reviewed, resulting in low reference credibility and verifiability scores. These factors allow for potential biases and false information, which pose a significant risk in medical education as students may rely on subjective, algorithm-driven content rather than high-quality evidence-based information. Additionally, with extended inquiry of the original prompt, this platform requires the user to purchase credits to continue the discussion. If a new prompt is created, it causes resetting the thread and a loss of conversational memory, forcing the user to re-establish the clinical context before continuing, disrupting the natural flow of the learning process. This is a major challenge as follow-up questioning after the initial query is essential for medical students to gain a deeper understanding of the material, especially in complex areas of study such as pharmacology. ChatGPT Plus was useful for creating a clear, patient-centered clinical overview, but it provided less depth than the platforms designed specifically for academic literature searching. ChatGPT had moderate credibility and verifiability scores, but most of the references provided were not peer-reviewed, suggesting that ChatGPT may better serve as a quick foundational guide to explain ideas and broad concepts, rather than as a primary research tool. A recent study assessing the accuracy of ChatGPT using clinical queries yielded similar results that although the platform is very accurate with factual knowledge, it does not perform as well when given more complex questions that require interpretation (Eskandar, 2026).

One objective of this study was to evaluate how AI incorporates the tenets of osteopathic medicine into its outputs, and we found that the level of integration varied by platform. In Queries 1 and 3, nearly all references provided by the three platforms had little to no osteopathic affiliation, whereas since Query 2 explicitly mentioned OMT, it predictably yielded predominantly osteopathic-affiliated references. In the assessment of OMT for chronic pain, both ChatGPT and Perplexity characterized OMT as a generic adjunct or alternative rather than an effective therapeutic strategy. Consensus Deep provided a more robust and academically grounded response, with references from osteopathic-associated authors or institutions. For Query 3, ChatGPT recognized several osteopathic themes but remained relatively surface-level compared to the depth provided by Consensus. In its 2026 United States Physician Workforce Data Dashboard, the Association of American Medical Colleges (American Association of Medical Colleges, 2026) reported that DO (Doctor of Osteopathic Medicine) physicians constitute 8.2% of total physicians, compared to the 65.2% of MD (Medical Doctor) physicians. Although PubMed has millions of citations in 2026, only a small fraction of this research comes from osteopathic medicine. Therefore, osteopathic medicine and its approach remain underrepresented in clinical practice and the literature, emphasizing the importance for learners to critically evaluate AI-generated information to ensure alignment with osteopathic philosophy and practice.

## Limitations, reflections, and future directions

A primary limitation of this study is the reliance on a specific snapshot of AI capabilities at the time this study was completed. The rapid growth of AI platforms and LLMs indicates that the data collected here will shift as platforms continue to develop, and models become more sophisticated. The same query can be input at different times within the same week and yield varying results, which complicates reproducibility. Additionally, platform memory may influence outputs differently depending on the AI platform being used. ChatGPT Plus appeared to be influenced by previous queries or context, whereas Consensus Deep generated responses independently and was not influenced by prior queries. This difference may have affected the consistency and comparability of responses across platforms.

The richer content returned for Query 1 (GLP-1 agonists) compared to Query 3 (geriatric polypharmacy) likely reflects the volume of internet content on GLP-1 agonists, which is currently a high-profile topic, rather than true platform capability. In addition, since Query 2 specifically asked for OMT, the results included an osteopathic approach, which was an expected result rather than a study finding.

Another limitation is the rapidly changing availability and recognition of AI platforms. As AI continues to develop, new platforms are frequently introduced; therefore, not all potentially relevant platforms were included in this study. For example, OpenEvidence was not included in this study since access is restricted to licensed healthcare professionals and medical students only (it excludes basic science faculty). As a result, the platforms evaluated in this study do not represent all available AI platforms, and future research may benefit from including medical-specific AI tools as they continue to emerge.

Furthermore, the use of these platforms is heavily reliant on proper prompt engineering to achieve high-quality outputs, meaning that the use of these tools depends on existing AI literacy. The research team was composed of first-year osteopathic medical students and a pharmacology faculty member, all of whom are familiar with using AI platforms but do not have formal training in prompt engineering. This creates a gap in which the quality of medical information generated is dependent on prior AI knowledge rather than on the platform database.

Another limitation is the potential cost barrier of each platform; Consensus tended to have better results, but the cost of $65 USD/month may be prohibitive. Perplexity Pro and ChatGPT cost $17 USD and $20 USD, respectively, which may be more affordable. Free versions of some platforms are available, but their capabilities are limited. As a result, a student’s experience on the platform may differ depending on whether they can afford a higher priced subscription tier. The ability to perform a direct head-to-head comparison of the three AI platforms also represents a limitation as each was developed to serve fundamentally different functions.

Additionally, Perplexity Pro utilizes a Model Council feature that incorporates multiple AI platforms, including ChatGPT, and selects the model best suited for the query input by the user based on task type, output needs, and available resources. This introduces further variability from prompt to prompt when using Perplexity. ChatGPT Plus also has various models to choose from, such as “Thinking,” “Extended,” and “Latest,” all having potential to affect response quality, length, depth, or level of detail, further adding variability to usage. Lastly, the opaque nature of the platform’s reasoning pathways complicates reproducibility as the logic used to generate the response to the query can change without notice to the user. It should be noted that each platform was explored to the same depth as another when we ran our queries to prevent confounding the results.

While analyzing the responses to our pharmacology-based queries provided by these different AI platforms, the variability in quality, reliability, and style of each was noteworthy. Students frequently use ChatGPT to aid in studying, particularly for generating practice questions, without considering whether the platform is best suited for the task. Perplexity Pro offered more detailed responses but often cited lower-credibility references, such as website posts and blogs, rather than the peer-reviewed scientific literature. In contrast, Consensus Deep, which was the most expensive, consistently provided sources with high credibility and verifiability scores, which is expected since it is a research-specific AI platform that specializes in providing users with research assistance in navigating the scientific literature.

From the perspective of an osteopathic medical student, the responses from each platform seemed to lack a true understanding of the osteopathic approach. This was not surprising, considering the limited osteopathic literature in training datasets that inform current AI models and LLMs (Newhardt et al., 2026). Although AI has the potential to be an extraordinary resource for medical information and a means of studying in medical education, there is no way for the platforms to form human connections with users; therefore, they have no capacity to truly grasp the human-relationship-centered approach used in practice by osteopathic physicians.

Another interesting observation was the significant imbalance in the quantity and quality of information across queries. All platforms provided more robust and detailed responses for Query 1 on GLP-1 receptor agonists for weight loss, compared to Query 3 on adverse drug reactions and geriatric polypharmacy, with substantially more references retrieved for Query 1 (data not shown). This reflects the widespread public interest and online attention surrounding GLP-1 receptor agonists for weight loss and diabetes, despite the significant health burden of polypharmacy in older adults. Perplexity Pro also frequently retrieved information from socially prominent but low-quality sources, such as social media posts and blogs, which raises ethical concerns. These findings highlight the importance of critically evaluating AI-generated information, especially when addressing vulnerable patient populations, as reliance on information from low-quality sources can risk patient safety and exacerbate health disparities.

As first-year medical students, we have transitioned from using traditional search engines such as Google to now using AI platforms to acquire knowledge and apply it to clinical scenarios. This study reinforced the need for critical appraisal of AI-generated information to avoid misinformation, promote patient safety, and maintain an osteopathic approach to patient care. Although holistic care is represented in the medical literature and in AI-generated responses, osteopathic tenets such as structure–function relationship or the body’s ability of self-healing were much less consistently represented, reflecting the limited availability of the osteopathic literature in AI training datasets. Based on these findings, we developed recommendations for using AI platforms as learning tools (Table 5). Ultimately, AI platforms in their current state can serve as valuable supplements to medical education and clinical practice, but they should complement rather than replace critical thinking and independent appraisal in technology-enhanced environments.

**TABLE 5:** Recommendations for using AI platforms as learning tools.

Tip	Rationale
Be specific with prompt input	Reduces inclusion of irrelevant information and increases chances of pertinent output
Revise prompts several times to generate the highest-quality results	AI may not understand what you are specifically looking for, and each prompt revision helps guide the AI platform to your desired results
“Train” the platform to a sufficient level first	By establishing context and specific criteria, AI platforms are more likely to generate pertinent, relevant, and desired output
Select the proper AI platform for your research goals	Different AI models excel at different tasks (Consensus is great for journal and evidence-based research outputs, whereas ChatGPT may give a good overall “birds eye” view of the topic)
Use the proper mode for your research goals in each platform	Each AI platform’s model excels in different tasks; “thinking” modes give a longer, more detailed response at the expense of time, whereas “fast” models give a surface level response faster
Use AI to supplement understanding, not as a sole learning method	Since AI occasionally hallucinates, it cannot be used as a primary source for information. However, it can excel with quick summaries and help guide and refine learning
Investigate where and how responses are being generated	Ensures that information quoted is from a reliable source, which helps minimize false information being spread
Appreciate that previous queries can influence later responses	Previous responses can bias future responses in the context of each conversation, which may affect desired results

## Data Availability

The raw data supporting the conclusions of this article will be made available by the authors, without undue reservation.
